# Pareto-Efficient Utilization of Coated Vermiculite Aggregate in High-Strength Lightweight Mortar with Mohr–Coulomb Parameter Analysis

**DOI:** 10.3390/ma18204652

**Published:** 2025-10-10

**Authors:** Zeynep Algin, Muhammed Şerif Yoluk, Halil Murat Algin

**Affiliations:** 1 Department of Civil Engineering, Harran University, Osmanbey Campus, 63000 Şanlıurfa, Turkey; zyilmaz@harran.edu.tr; 2 Technical Sciences Vocational School, Harran University, Eyyübiye Campus, 63000 Şanlıurfa, Turkey; serifyoluk@harran.edu.tr

**Keywords:** lightweight mortar, expanded vermiculite, multilayer coating, Pareto-efficiency analysis, Mohr–Coulomb parameters, shear strength

## Abstract

A multilayered coating process, based on cement and silica fume, was applied to the surface of expanded vermiculite aggregate (EVA) using a cold bonding method. This investigation represents the first systematic study of this multilayered coating method, with the objective of evaluating the effectiveness of coating thickness in the production of high-performance lightweight mortar. In the experimental phase of this study, a range of aggregate replacement levels was examined, and a series of tests were conducted to assess parameters such as dry density, porosity, thermal conductivity, water absorption, sorptivity, compressive strength, flexural strength, and shear strength. The obtained Mohr–Coulomb (MC) constitutive model parameters and shear strength properties were verified numerically. The verification process facilitated the simulation of the three-dimensional (3D) combined behavior of the produced mortar with cement paste, cement–silica fume liner, and EVA. The simulation was conducted using a micro-scale finite element (FE) model based on the Computer Tomography (CT) data. The Pareto-efficient utilization boundaries of coated-EVA in the production of high-strength lightweight mortar are then specified using Response Surface optimization analyses. The present study demonstrates that the cold bonding multilayered coating process is a highly effective aggregate-strengthening method. This study revealed that the Pareto-efficient replacement range of coated-EVA is 24–58%, corresponding to a coating thickness of 0.9–2.6 mm. It is evident that the effective utilization of the replaced aggregate in the mortar production is subject to a limit, which can be determined through Pareto-efficiency analysis, and it is contingent upon the performance requirements of the resulting mortar.

## 1. Introduction

The construction sector has recently accorded increasing attention to lightweight mortars, which offer a number of specific benefits. These include excellent thermal and acoustic insulation [1,2]. The utilization of lightweight aggregates of natural and man-made origin presents considerable economic and environmental advantages, characterized by a reduction in thermal conductivity and density in comparison to traditional aggregates [3,4]. The use of natural aggregates has caused controversy owing to their excessive use in many regions and the scarcity of natural resources [5,6]. Consequently, the utilization of synthetic lightweight aggregates in the construction industry is experiencing a notable surge [7,8]. Artificial lightweight aggregates are produced through techniques such as sintering and cold bonding. Sintered expanded clays, expanded shale, and fly ash aggregates, produced via heat treatment at 1000–1300 °C, are commonly used in lightweight mortars [5]. However, sintering is highly energy-intensive and emits large quantities of pollutants [9].

Conversely, cold bonding satisfies environmental and economic criteria due to its lower energy demand. Many recent studies have focused on lightweight aggregate production using cold bonding [7,8]. These aggregates typically have greater density than sintered ones. Therefore, Tajra et al. (2018) [10] developed low-density lightweight aggregates by encapsulating expanded perlite using cold bond granulation, a core–shell method. In another study, Tajra et al. (2018) [11] used these aggregates in concrete, evaluating properties like consistency, dry density, thermal conductivity, water absorption, and compressive strength. The results showed that these aggregates are suitable for producing structural lightweight concrete. The current study advances single-layer coating (e.g., [10,11,12]) by applying a multilayer process, producing aggregates with significantly improved strength. This was implemented on EVA to achieve the desired strength. This study utilizes the concept of aggregate engineering to enable designer-defined improvement via multilayer coating.

Low thermal conductivity, bulk density, heat resistance, chemical inertness, and environmental friendliness make expanded vermiculite (EVA) suitable for construction [13]. Many studies have examined vermiculite in mortar [14,15,16,17]. Mo et al. (2018) [16] noted that its porous structure reduces compressive strength and unit weight by increasing water absorption. At 30–60% EVA replacement, density values were 1774–1485 kg/m^3^—a 12–27% reduction—while compressive strength declined 50–63%, to 16.6–12.1 MPa [16]. Tie et al. (2022) [17] reported 24% and 50% density reductions at 50% and 100% replacement. Compressive strength fell to 9.7 and 3.1 MPa from a control value of 48.1 MPa [17]. Koksal et al. (2015) [15] investigated EVA/cement ratios of 4–8% and silica fume additions of 0–15%, reporting unit weight, water absorption, and compressive and flexural strengths as 780–1200 kg/m^3^, 24.2–40.6%, 5.8–16.4 MPa, and 2.1–4.8 MPa, respectively. Thermal conductivity fell to 0.257 W/mK, with thermal performance improving by 58.2% [15].

Although EVA allows for low-density mortars, its use significantly reduces mechanical strength [15,16,17]. This challenge is addressed here through a multilayer strengthening process with EVA, a first in the literature. Thus, a more rational coating process is established for lightweight mortar production. Moreover, structural designers often rely on assumed values for Mohr–Coulomb parameters when modeling lightweight mortars with EVA, compromising accuracy. Hence, this study also determined Mohr–Coulomb strength parameters for mortars incorporating coated-EVA. These data enable more accurate modeling of mortar shear behavior and enhance the application of material definitions in finite element simulations. Additionally, a pragmatic design scheme is proposed, optimizing coating thickness and aggregate replacement ratio based on targeted performance. The Pareto-optimal method, in conjunction with response surface methodology (RSM), was employed in the present study. The Pareto-optimization approach is employed in the context of multi-objective optimization problems, given the understanding that the resulting solution does not exhibit a single global optimum and that the decision maker possesses multiple alternatives. Pareto-efficiency can be delineated as a set of solutions wherein the attainment of improvement in one objective function is mutually exclusive with the worsening of other objective functions [18]. This feature has been demonstrated to provide more realistic and applicable solutions in comparison to conventional single-objective optimization techniques [18]. Accordingly, the efficiency analysis guarantees a balanced evaluation of the relative performance of decision units according to several criteria [19].

## 2. Multilayer Strengthening Process of EVA

A review of the literature reveals that a limited number of materials and aggregate types have been subjected to a single-stage coating process by cold bonding using cement and pozzolan [10,11,12,20,21,22,23,24,25]. Although cold bonding, a similar aggregate production process used in the literature, was also employed in this study, the coating thickness was fixed in all of the aforementioned studies [10,11,12,20,21,22,23,24,25,26,27,28,29]. In contrast to the present research, the coating process in these studies was implemented as a single layer due to the production technique utilized. However, the multiple-coating approach described in this research allows the strength of the aggregate to be regulated or customized according to the required strength through the thickness of the surface coating in the multilayer coating process. This method allows the aggregate to meet specific requirements. Accordingly, the production process can be conducted in accordance with the optimal coating thickness, thereby achieving the desired strength or responses, such as density and thermal conductivity, in mortar production.

In this context, the quantity of cement paste can be modified in accordance with the desired strength and density of the lightweight mortar, thereby ensuring that the coated-EVA is produced with the requisite qualities and attributes for mortar design and the coating thickness necessary to satisfy the specified parameters. This approach allows for the formulation of more effective mortar production, which can be achieved by utilizing aggregates designed explicitly during the aggregate production stage. Accordingly, the implementation of aggregate engineering has the potential to enhance mortar production in the construction industry.

**Figure 1:**
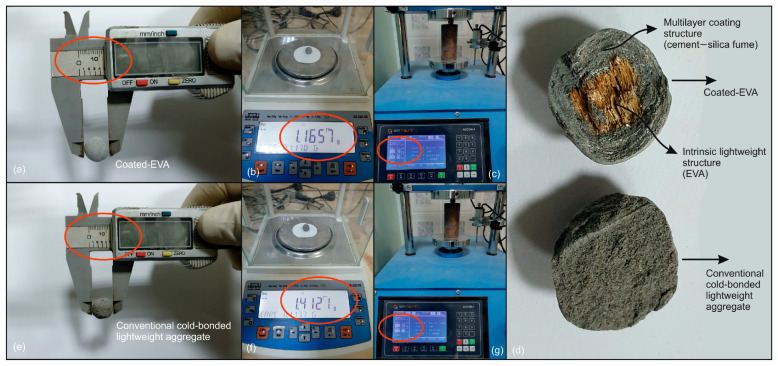
Comparative analysis of coated-EVA and cold-bonded pellet aggregates with representative measurements marked with red circle: (**a**,**e**) diameter, (**b**,**f**) mass, (**c**,**g**) uniaxial failure load, and (**d**) close-up internal views.

Figure 1 presents a comparison of the mass and fracture load of a coated-EVA and a similar-sized conventional lightweight pellet aggregate made using cold bonding. The coated-EVA, with a diameter comparable to that of a conventional pellet aggregate, exhibits a notable reduction in weight and an enhanced fracture load capacity (Figure 1).

**Figure 2:**
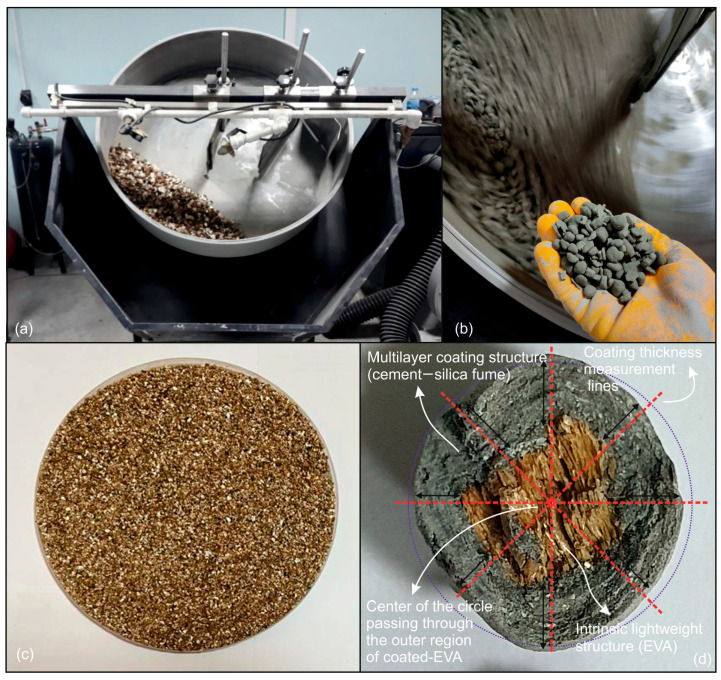
Coating process of EVA and measurement of average multilayer thickness: (**a**) pelletizing device, (**b**) coating process, (**c**) raw EVA, and (**d**) close-up of a coated-EVA particle with thickness measurement planes.

**Figure 3:**
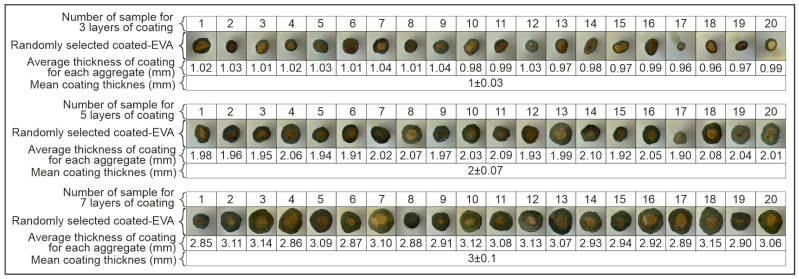
The process of aggregate thickness calculation.

In the preliminary stage of this research, coating thicknesses of approximately 1 ± 0.03 mm, 2 ± 0.07 mm, and 3 ± 0.1 mm—corresponding to three, five, and seven successive coating applications, respectively—were investigated. For the sake of simplicity, these were nominally referred to as 1 mm, 2 mm, and 3 mm in subsequent sections, with further procedural details provided below. Preliminary laboratory studies have demonstrated that the coating of EVA with a thickness greater than 3 mm frequently results in the material falling outside the permissible density limits defined by ASTM C330 [30] for lightweight aggregates. Accordingly, a maximum acceptable coating thickness of 3 mm was established in order to ensure compliance with the standard specifications. Consequently, coating applications resulting in thicknesses beyond this threshold were excluded from the experimental scope of this study. During the aggregate production process, once the targeted number of coatings has been achieved, random samples are taken from each production batch, with a minimum of 20 samples per batch. The thickness of the coating for each aggregate is measured, as illustrated in Figure 2, and the standard deviation of these values is calculated. Given that all standard deviations were found to be less than 5%, it was possible to determine the coating thickness corresponding to the number of layers with a high degree of confidence. Consequently, the mean value of the coated aggregates, along with the standard deviation value, was considered. The thicknesses corresponding to 3, 5, and 7 layers of coating were measured as 1 ± 0.03 mm, 2 ± 0.07 mm, and 3 ± 0.1 mm, respectively. The process of aggregate thickness calculation is illustrated in Figure 3. The standard deviation values were omitted from this article for practical reasons. It is evident that the median values between the sample measurements are consistent with each other. This finding suggests that the production process was executed in a homogeneous manner. Consequently, the production continued as outlined in the methodology section.

The coating process of the EVA is illustrated in Figure 2. The EVA, comprising a very fine fraction sourced from the Karakoç Mine in the Yıldızeli district of Sivas in Turkey, was pelletized with a cement/silica fume paste. It was assumed that 15% silica fume would be used in the coating of the EVA. The 15% silica fume content was considered as the lower bound of the optimum range suggested by several researchers (e.g., [31]) and was adopted in the present study. The initial coated aggregates were subjected to membrane curing (20 °C temperature and 70% humidity) for a period of 24 h. Subsequently, the aggregates were pelletized once more in order to form the second layer. The pelletized aggregates were subjected to a second period of membrane curing, after which they were pelletized once more to form the third layer. This process was repeated until the seventh layer was reached. The coating process was terminated after seven layers, as it was deemed that the resulting density of the aggregates and mortar would exceed the lightweight limiting values. The coated-EVAs with three, five, and seven layers were subjected to membrane curing for a period of 28 days.

In the strengthening process of EVA, aggregates with homogeneous properties were produced by the cold bonding method. This ensured the use of the pelletizing device at a standard angle and speed, and working with standard material quantities in production. Figure 2d depicts a representative image of coated-EVA, wherein the coating thickness is quantified in four directions and the mean diameter of the coated-EVA is ascertained during the production process. Furthermore, the aggregate grain size distribution (ASTM C136 [32]), loose bulk density (ASTM C29 [33]), water absorption, and specific gravity values (ASTM C128 [34] and ASTM C127 [35]) were also determined for the aggregates produced.

## 3. Materials and Methods

**Figure 4:**
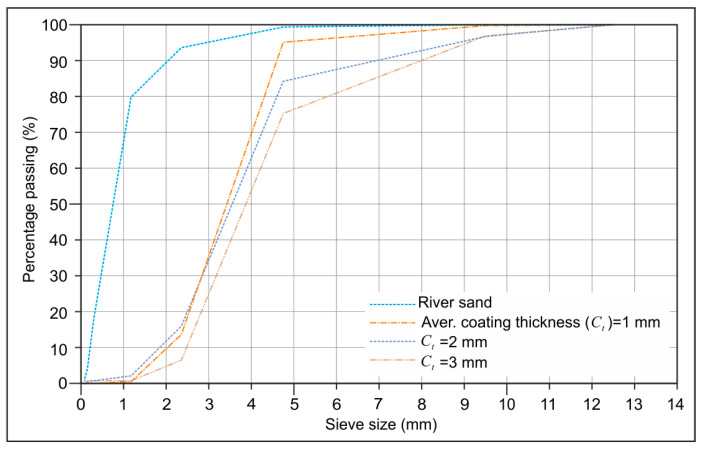
Gradation curve of natural sand and coated-EVA.

CEM II/A-M (P-LL)-42.5 N cement type, with a specific gravity of 3.06 and a Blain fineness of 4085 cm^2^/g by the EN 197-1 standard [36], was employed in conjunction with silica fume, with a specific gravity of 2.2 and a Blain fineness of 200,000 cm^2^/g, obtained from Antalya Eti Elektrometalurji Inc., Antalya, Turkey. River sand with a specific gravity of 2.63 was employed as a normal-weight aggregate in mortar mixtures. The gradation curves of river sand and coated-EVA with three distinct coating thicknesses produced for this study are presented in Figure 4.

As illustrated in Table 1, the specific gravity values of coated-EVA exhibit a range of 1.75–1.81, while the loose bulk density falls within the 360–379 kg/m^3^ range. Additionally, the water absorption values of coated-EVA vary between 18 and 24%, contingent upon the average coating thickness. Given that the grain density and bulk density of coated-EVA are less than 2000 kg/m^3^ and 1200 kg/m^3^, respectively, the aggregates satisfy the criteria for lightweight aggregates as defined in EN-13055 [37]. In this study, 12 mortar mixes were produced by substituting river sand with coated-EVA, with replacement ratios of 33%, 66%, and 100% for each of the four different coating thicknesses. The quantities of materials employed in the mortar mixtures are presented in Table 2. During sample preparation, the fresh mortar was compacted using a vibration table in accordance with ASTM C192 [38]. After 24 h, the specimens were demolded and subjected to water curing until the designated testing age.

The consistency of fresh hydraulic cement mortar was determined in accordance with ASTM C1437 [39]. Fresh mortar was placed into the cone mold in two layers, each compacted by tamping 20 times with a standard tamping rod. After leveling the surface flush with the top of the mold, the cone was carefully lifted vertically. Within 1.5 min of filling the mold, the table was dropped 25 times in 15 s. Following the drops, the resulting spread diameter of the mortar was measured in two perpendicular directions, and the average value was reported as the flow diameter. The oven dry density, apparent porosity, and water absorption tests were performed in accordance with the standards set forth in ASTM C642 [40]. The experiments were conducted using 28-day specimens with dimensions of 50 mm× 50 mm× 50 mm. The water absorption test was carried out in accordance with this standard. Specimens were first oven-dried at 105 ± 5 °C to a constant mass, then immersed in water at 20 ± 2 °C for 24 h, after which the saturated surface-dry mass was recorded. The compressive strength test was conducted in compliance with the ASTM C109/C109M standard [41], utilizing a 3000 kN capacity press. The test was conducted on the prepared specimens with dimensions of 50 mm× 50 mm× 50 mm on the 28th day. The flexural strength test was performed according to the ASTM C348 [42] standard. The test was conducted on the prepared prism specimens, measuring 40 × 40 × 160 mm, on the 28th day. A capillary water absorption test was conducted according to the ASTM C1585 standard [43]. The test was carried out on 28-day specimens measuring 50 × 50 × 50 mm. In this experiment, the initial rate of absorption was determined using measurements made within the first 6 h, while the secondary rate of absorption was determined using measurements made between 1 and 8 days. The initial and secondary absorption rates were calculated by considering the slope of the regression curve derived from the relationship between the square root of the elapsed time and the amount of water absorbed per unit area. A thermal conductivity test was conducted on two specimens of lightweight mortar, each measuring 50 mm in length, width, and height. Prior to testing, the specimens were oven-dried. The thermal conductivity coefficient was determined in accordance with the ISO 22007-2 standard [44] using a TPS 500 S Hot Disk Thermal Constants Analyzer.

**Figure 5:**
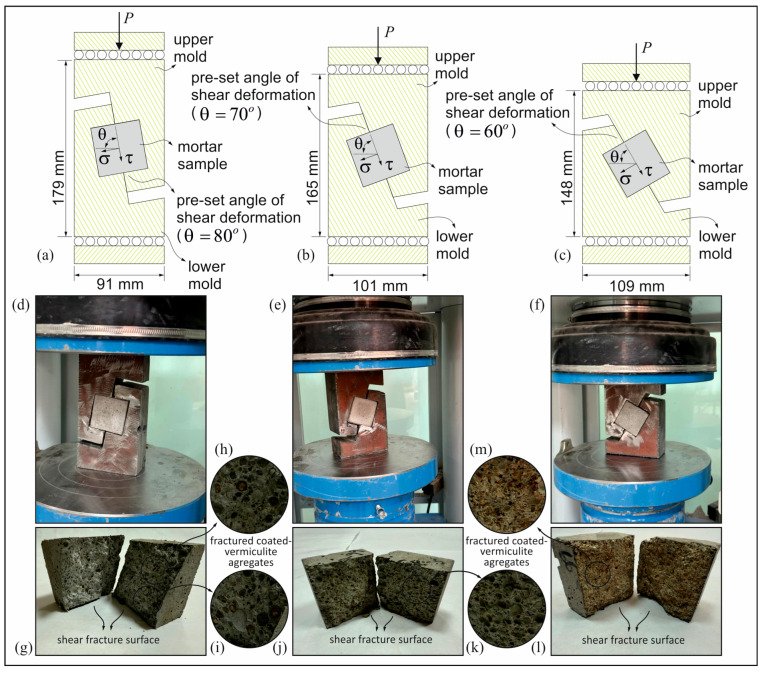
The pre-set angular shear test setups for the mortar samples and the shear fracture surfaces. The pre-set deformation angles are set at 80°, 70°, and 60° with respect to the normal stress (**a**–**c**), and the schematic illustration of the mold system (**d**–**f**), respectively. The shear fracture surface photographs from the pre-set angular shear tests (**g**–**m**).

In defining the MC constitutive material model, it is essential to have accurate knowledge of the material’s cohesion and friction angle parameters. In this context, the system used in the pre-set angular shear test is shown in Figure 5a–f. As shown in Figure 5a,d, the pre-set deformation angle is set at 80° with respect to the normal stress. However, the mold system shown in Figure 5b,e has a pre-set deformation angle of 70°. The mold system in Figure 5c,f has a pre-set deformation angle of 60°. It is evident that the pre-set deformation angles of these molds are fixed and positioned close to the shear stress axis (Figure 5a–c). This results in fractures occurring at lower compressive loads. The main advantage of this particular mold system is that the process can be carried out using a standard concrete press. The development of this mold system for cubic concrete specimens with dimensions of 100 × 100 × 100 mm was carried out by Lelovic and Vasovic, (2020) [45], and subsequent mechanical verification was carried out. In this study, for each pre-set angular deformation, three specimens of 50 mm× 50 mm× 50 mm from each mix were tested and averaged. The photographs of the pre-set angular shear tests are shown in Figure 5, which shows that the shear failure surfaces are compatible with the pre-set angle of the mold system. The shear and normal stress values at the shear failure surface of the specimen were calculated as a function of the pre-set deformation angle using the compressive load (P) applied to the mold system.

## 4. Results and Discussion

**Figure 6:**
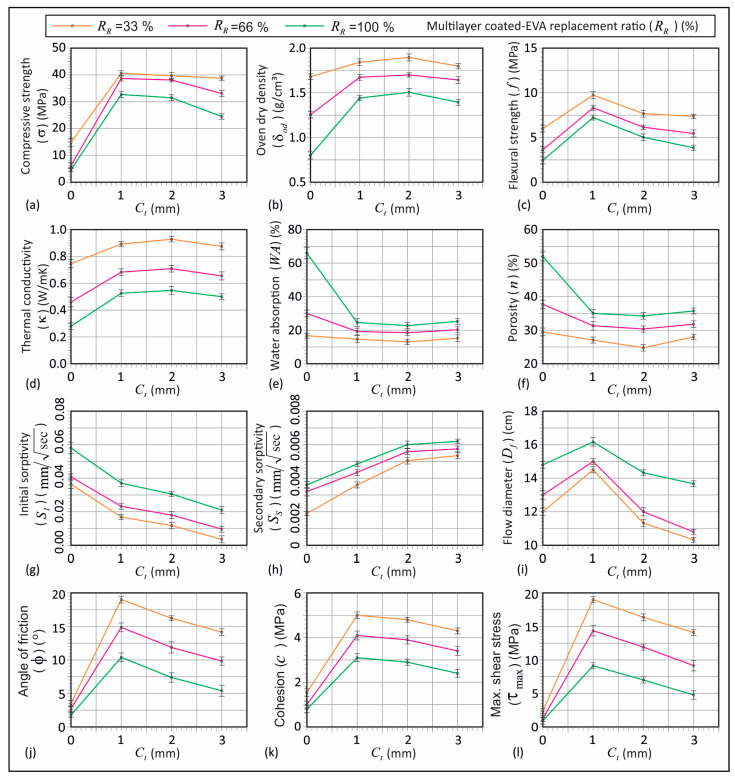
Test results and the error bars with standard deviation: (**a**) compressive strength, (**b**) dry density, (**c**) flexural strength, (**d**) thermal conductivity, (**e**) water absorption, (**f**) porosity, (**g**) initial sorptivity, (**h**) secondary sorptivity, (**i**) flow diameter, (**j**) angle of friction, (**k**) cohesion, and (**l**) maximum shear stress.

Figure 6 presents the results of the tests performed on the lightweight mortar mixtures produced with 33%, 66%, and 100% replacement ratios (*R_R_*) of coated-EVA with various coating thicknesses (*C_t_*), including the error bars with standard deviations. The tests included compressive strength, oven dry density, flexural strength, thermal conductivity, water absorption, apparent porosity, initial and secondary absorption rate, flow diameter, and pre-set angular shear.

Figure 6a shows the impact of replacement ratios and coating thicknesses on compressive strength. An increase in the replacement ratio reduced compressive strength across all coating thicknesses. The lowest value (4.7 MPa) occurred in mortar with *R_R_* = 100% uncoated-EVA (*C_t_* = 0). Coated-EVA improved compressive strength for all ratios. Strengths for *R_R_* = 33% and 66% at *C_t_* = 1 mm and 2 mm were similar (~40 MPa). Uncoated-EVA at 33% and 66% yielded ~15 MPa and 6 MPa, respectively. *C_t_* = 3 mm caused a slight strength reduction. The highest strength occurred with a 1 mm coating at all ratios (Figure 6a). For *R_R_* = 100%, coatings of 1, 2, and 3 mm increased strength by ~600%, 570%, and 420%, respectively, over uncoated-EVA. The literature shows that uncoated-EVA significantly lowers strength [15,16,17,46]; values at 30–100% substitution were 3.1–16.6 MPa [16,17]. This is due to EVA’s porous, malleable structure [47], which weakens load-bearing capacity and crack resistance [16].

An increase in coating thickness directly increases the effective diameter of aggregate particles, thereby altering the particle size distribution within the mortar. Increases in aggregate particle size, particularly at high coating thicknesses (e.g., 3 mm), result in a relative decrease in the bond surface area between the aggregate and the cement matrix. This, in turn, has a detrimental effect on the behavior of the composite material, resulting in a reduction in mortar strength, as shown in Figure 6a,c. This phenomenon has also been observed in concrete with different aggregate changes (e.g., [48,49,50]). Moreover, the presence of large-sized coated particles has been demonstrated to disrupt the homogeneity of the mortar, leading to localized stress concentrations on the surfaces of the aggregate. This has the potential to compromise load transfer efficiency, consequently leading to diminished compressive and flexural strength, as shown in Figure 6a,c. Consequently, an augmentation in particle size, engendered by an increase in coating thickness, has the potential to adversely impact aggregate–matrix interaction, thereby diminishing mechanical performance. This behavior has also been observed in conventional concrete (e.g., [48,49,50]). This finding, consistent with the results obtained, highlights the importance of maintaining the coating thickness at an optimal level. In mortars incorporating multilayer coated-EVA aggregates, the aggregate core retains the porous structure of the raw EVA, which inherently influences the mechanical response by enhancing compressive strength while limiting flexural strength.

**Figure 7:**
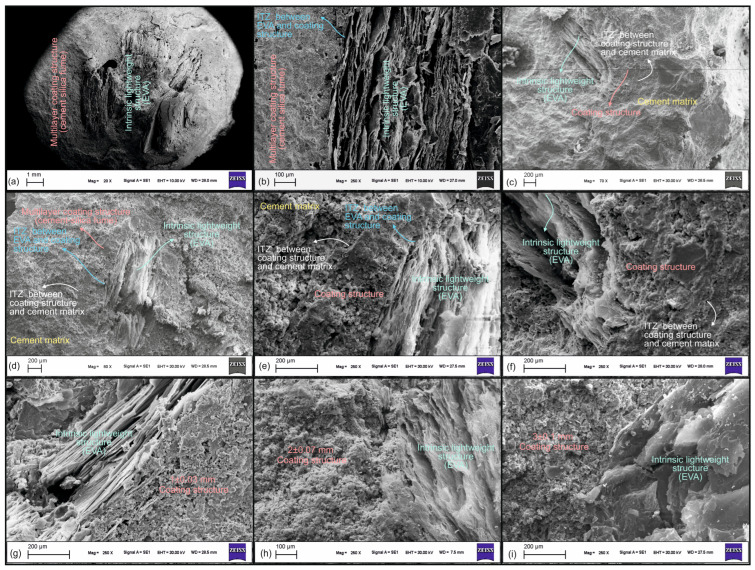
SEM images of coated-EVA and the produced lightweight mortar: (**a**) close-up view of coated EVA, (**b**) ITZ between EVA and the coating structure, (**c**) ITZ between the coating structure and the cement matrix, (**d**–**f**) ITZ views of coated EVA aggregates and the cement matrix in mortar, and (**g**–**i**) ITZ views between EVA and the coating structure for various coating thicknesses.

Figure 7 shows SEM images of coated-EVA and mortar. SEM reveals hollow vermiculite, dense coating, and ITZ between the coating and EVA. Also shown is ITZ between coated-EVA and the cement matrix. Coating weak vermiculite with dense layers creates a stronger ITZ with cement, improving overall structure and resisting fracture.

Figure 6b illustrates oven dry density variations with different coating thicknesses. The lowest values for 33%, 66%, and 100% R_R_ were 1.68, 1.26, and 0.79 g/cm^3^ for uncoated-EVA. The literature reports similar values: 1.11–1.77 g/cm^3^ for 30–100% EVA [16,17]. Coated-EVA increased density to 1.4–1.89 g/cm^3^. For all ratios, a thicker coating led to higher density, as coated-EVA has a greater specific gravity. With a 2 mm coating, the highest oven dry density was recorded. For 33% *R_R_*, densities at 0, 1, 2, and 3 mm were 1.68, 1.84, 1.89, and 1.80 g/cm^3^, respectively. Since all values were <2 g/cm^3^, coated-EVA is viable for lightweight mortar.

**Figure 8:**
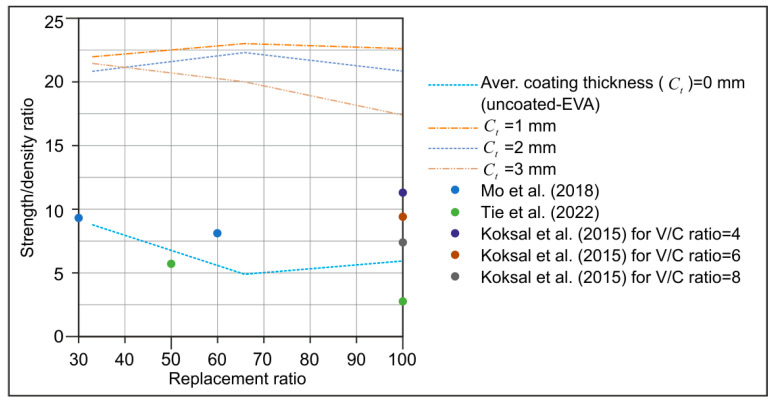
Comparison of strength/density ratios between the coated-EVA in this study and those reported in the literature [15,16,17].

Figure 8 illustrates the correlation between the coated-EVA replacement ratios and the strength/density (s/d) ratios. The s/d ratio of the specimens with uncoated-EVA exhibits a range of 4.9 to 8.8. The literature is also shown in Figure 8 as in the range of 2.8–11.3 [15,16,17,46]. Coated-EVA, however, significantly boosted strength with a modest density increase due to its structure. Coated-EVA mortars had s/d ratios of 17.5–23.1. This method enhances strength without compromising density, essential for lightweight mortar.

Figure 6c illustrates the variation in flexural strength obtained from samples with coated-EVA replacement ratios of 33%, 66%, and 100% in terms of coating thickness. An increase in the replacement ratio of EVA is associated with a reduction in the flexural strength of the mortar. The lowest flexural strength values were 6.04, 3.63, and 2.44 MPa for mortar specimens with uncoated-EVA at 33%, 66%, and 100% replacement ratios, respectively. As observed in previous studies, flexural strength values for mortar samples with uncoated-EVA fall within the range of 2.1–4.8 MPa [15]. Figure 6c shows that the lowest flexural strength was 2.44 MPa in the mortar with uncoated-EVA at 100% replacement, while the highest was 9.76 MPa in samples with coated-EVA at 1 mm thickness and 33% replacement. Coated-EVA enhances flexural strength at all replacement ratios compared to uncoated-EVA. Specimens with a 1 mm coating exhibited the highest strength across all ratios. Flexural strengths of specimens with 33%, 66%, and 100% coated-EVA at 1 mm thickness were approximately 61%, 130%, and 197% higher, respectively, than those with uncoated-EVA.

Figure 6d shows the variation in thermal conductivity of coated-EVA with the replacement ratio and coating thickness. An increase in the replacement ratio decreases the thermal conductivity of mortar, regardless of coating thickness. At 100% replacement, thermal conductivity values for 0, 1, 2, and 3 mm coating thicknesses were 0.283, 0.526, 0.548, and 0.5 W/m·K, respectively. The lowest value was observed in mortars with uncoated-EVA. Thermal conductivity for uncoated-EVA at 33%, 66%, and 100% replacement was 0.747, 0.463, and 0.283 W/m·K, respectively. Similar to this study, previous research found thermal conductivity of mortars with vermiculite ranged from 0.257 to 0.633 W/m·K [15]. Figure 5d shows that the conductivity values rise up to 2 mm thickness, then slightly decrease at 3 mm. For a 2 mm coating thickness, values at 33%, 66%, and 100% replacement were 0.929, 0.710, and 0.548 W/m·K, respectively.

Figure 6e shows the correlation between water absorption and both average coating thickness and coated-EVA replacement ratios. Higher replacement ratios lead to increased water absorption across all coating thicknesses. The highest absorption is observed in uncoated-EVA samples. Absorption values at 33%, 66%, and 100% replacement for uncoated-EVA were about 17%, 30%, and 66%, respectively. Similar findings are reported in the literature [15,16,51,52]. Figure 6e indicates that coated-EVA significantly reduces water absorption at all ratios. However, changes in coating thickness have a negligible effect on water absorption. The lowest value, ~14%, was obtained at 33% replacement. Absorption values for 66% and 100% were approximately 19% and 24%, respectively.

Figure 6f shows the correlation between porosity and coating thickness with coated-EVA replacement. Higher EVA ratios lead to increased porosity regardless of coating thickness. The highest porosity was found in uncoated-EVA samples across all ratios. Porosity for 33%, 66%, and 100% uncoated-EVA samples was about 30%, 38%, and 52%, respectively. Koksal et al. (2015) [15] also reported porosity rising with vermiculite between 29 and 43%. Figure 6f demonstrates that coated-EVA reduces porosity at all ratios. However, changes in coating thickness have a limited effect. The lowest porosity, ~25%, was observed at 33% replacement and a 2 mm coating.

Figure 6g,h show the effects of the coating thickness and coated-EVA ratio on initial and secondary water absorption. Higher replacement ratios increased both absorption rates across all coating thicknesses. Initial absorption for 33%, 66%, and 100% was in the range of 0.0036–0.0364, 0.00965–0.0408, and 0.02095–0 mm/√s, respectively. Secondary absorption was 0.0019–0.00535, 0.0032–0.00575, and 0.0036–0.0062 mm/√s, respectively. Increasing coating thickness reduced initial but raised secondary absorption. Uncoated-EVA mortars had significantly higher porosity (see Figure 6f). Higher initial absorption and lower secondary absorption in uncoated-EVA mortars are due to greater porosity and rapid capillary absorption in the first six hours. As secondary absorption occurs later, capillary water intake decreases, resulting in lower secondary rates than those in mortars with coated aggregates.

Figure 6i illustrates the variation in flow diameter of mortar in relation to coating thickness and coated-EVA replacement ratios. The maximum flow diameter across all coating thicknesses ranges between 13.5 and 16 cm for 66% replacement. Overall, the maximum flow is between 14.5 and 16 cm for a 1 mm thickness. Mixtures with 2 mm and 3 mm coating thicknesses showed a notable reduction in flow.

**Figure 9:**
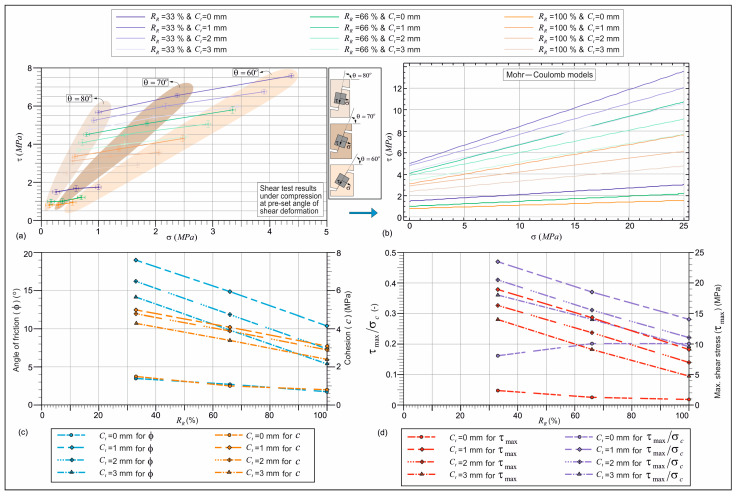
(**a**) The results from the pre-set angular shear test and the error bars with standard deviation; (**b**) MC constitutive material models; (**c**) effect of coated-EVA aggregate utilization on cohesion and friction angle values; (**d**) variation in $\tau_{\max}$ values.

Figure 9a demonstrates the results from the pre-set angular shear test, including the error bars with standard deviations. Mortar molds had pre-set deformation angles of 80°, 70°, and 60°, as shown in Figure 5. A decrease in angle increases average shear and normal stress values on the fracture surface. This shift displaces the fracture surface from the vertical axis, requiring higher compressive loads for failure. Figure 9b shows the MC constitutive models. The intersection with the shear stress axis corresponds to cohesion (*c*), and the slope represents the friction angle (ϕ). Figure 9c shows the effect of coated-EVA on *c* and ϕ, both of which decrease as uncoated and coated-EVA replacement ratios rise.

As shown in Figure 9c, ϕ for mortars with uncoated-EVA (33–100%) varies between 1.76° and 3.49°, while for coated-EVA, it ranges from 5.40° to 19.01°, depending on the coating thickness. Figure 6j demonstrates that coated-EVA increases ϕ. A coating strengthens the EVA surface, limiting deformation and increasing internal friction resistance. ϕ increased by 306–445% for 33% replacement, 261–447% for 66%, and 207–490% for 100%, compared to uncoated-EVA. Regardless of the replacement ratio, friction angle improvement declined with increasing coating thickness. Internal friction resistance depends on aggregate morphology, surface properties, and particle size [53,54]. The circular, smooth-coated aggregates reduced mechanical interlocking. Larger particle diameters decreased contact surface per volume, facilitating movement.

Cohesion values for mortars with uncoated-EVA (33–100%) ranged between 0.79 and 1.48 MPa (Figure 9c). Coated-EVA mortars had cohesion values from 2.45 to 5.01 MPa. Coated-EVA improved mortar cohesion by 190–238% (33%), 251–321% (66%), and 212–299% (100%), compared to uncoated-EVA. Figure 6k shows cohesion declines with increasing coating thickness. Thicker coatings enlarge particle size and reduce specific surface area, weakening paste–aggregate bonding. Difficulty in homogeneous coarse aggregate distribution can cause voids and reduce matrix integrity.

With uncoated-EVA, cohesion dropped 34% when increasing from 33% to 66%, and 47% from 33% to 100%. With coated-EVA, cohesion dropped 19% and 40% for the same replacements. These results confirm that coating thickness improves EVA’s mechanical performance.

Using shear strength parameters from Figure 9b and compressive strengths from Figure 6a, the maximum shear stress was derived and normalized (Figure 9d). For uncoated-EVA (33–100%), the maximum shear stress varied from 0.93 MPa to 2.39 MPa, while coated-EVA ranged from 4.77 MPa to 19.01 MPa. A coating significantly enhanced shear strength. Shear strength increased from 490 to 696% (33%), 622 to 1034% (66%), and 413 to 883% (100%) versus uncoated-EVA. Increasing coating thickness reduced maximum shear stress, but values for coated-EVA remained higher than those for uncoated across all thicknesses. The highest shear strength of mortar occurred with 1 mm coated-EVA.

## 5. Numerical Verification

**Figure 10:**
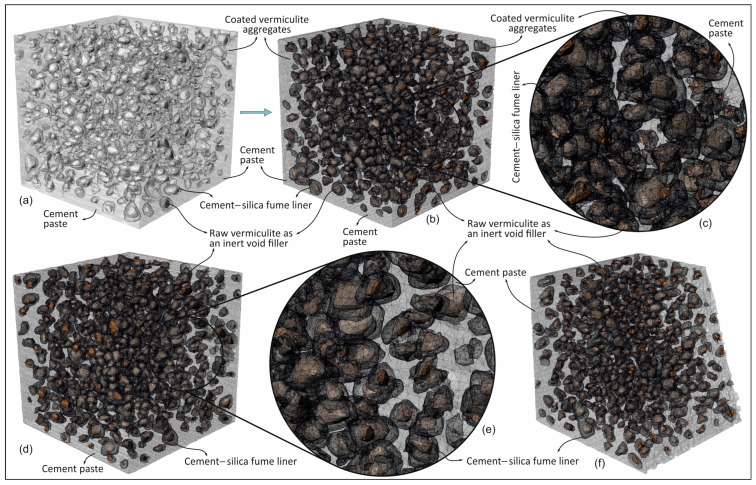
(**a**) The point cloud obtained as a result of CT scanning, and (**b**–**f**) images of the three-component (i.e., coated-EVA, cement paste, and ITZ) micro-scale FE mesh model of the mortar specimen.

In order to verify the pre-set angular shear test results (Figure 5 and Figure 10), a specimen was selected from the lightweight mortar samples that was made with coated-EVA aggregate (*R_R_* = 66%, *C_t_* = 1 mm). Prior to the execution of the pre-set angular shear test, a CT scan was conducted on the designated specimen. During the scanning process, utilizing the Philips Incisive CT clinical scanner, X-ray values were set to 120 kV and 200 mA, and a resolution of 0.3 × 0.3 × 0.3 × 0.3 mm/pixel was selected to enhance the efficacy of the boundary detection technique. The micro-tomography image scan results obtained were processed using the boundary detection technique in the Matlab (V.12a) (MathWorks, Natick, MA, USA) environment (Figure 10a).

In order to circumvent the intricacies associated with FE model analysis, the micro-model was filtered to include solely the coated vermiculate aggregate, cement paste, and the ITZ zone connecting these two phases. The ITZ zone was defined by the Coulomb contact model in the FE analysis. Figure 10a illustrates the filtered point cloud of the specimen obtained from CT scanning. It is acknowledged that modeling mortar with these three components (i.e., coarse aggregate, cement paste, and ITZ) can accurately reflect its mechanical behavior, as demonstrated by previous researchers (e.g., [55,56,57]). By modeling these components as a composite material at the micro-scale, the heterogeneous structure in mortar can be modeled realistically (Figure 10). During this process, the boundary detection process was carried out in accordance with the amount of coarse aggregate in the relevant mixture by means of the image processing method. FE mesh models were obtained using the obtained boundary structure (see Figure 10b–f). The utilization of non-destructive digital modeling of concrete using CT scanners has recently emerged as a highly popular approach to facilitate comprehension of the mechanical behavior of concrete and to discern its intrinsic components (e.g., [58,59,60,61,62,63]).

**Figure 11:**
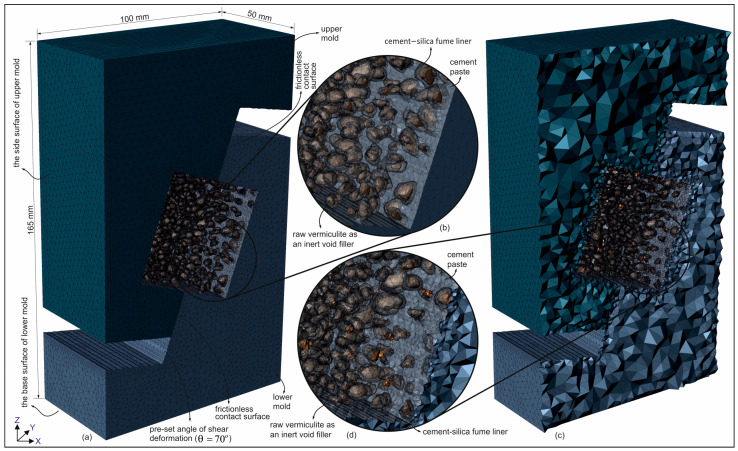
FE mesh modeling with the micro-scale specimen model with 70° pre-set angular shear test molds: (**a**) 3D-FE mesh, (**b**,**d**) close-up view of the mesh, (**c**) internal elements within the mesh.

The CT-derived 3D mesh was generated using tetrahedral elements, with a maximum element edge length of 5 mm and a minimum element size of 0.1 mm, resulting in approximately 1.7 million elements in total. A mesh sensitivity analysis confirmed that additional refinement did not significantly improve the simulation accuracy; therefore, this mesh density was adopted for all FE analyses. The boundary conditions were specified for the base surface of the lower mold (Figure 11) that was fully fixed in all directions, while for the upper mold, only the side surface in the y-direction (Figure 11) was constrained, and the remaining surfaces were left free to simulate realistic loading conditions. Contact interactions were limited to the interface between the upper and lower molds (Figure 11), which was modeled as frictionless, consistent with common assumptions used in laboratory loading setups.

In the continuum FE analyses, the coated-EVA and cement paste phases were first defined using the MC constitutive model. The FE mesh model of the test setup was constructed using the 70° pre-set angular shear test specimen (see Figure 5b,e). Subsequent analyses were performed using this model. The lower mold’s base is fully anchored, while the upper mold is constrained to be free of movement on the sliding surface (see Figure 11).

The experimental findings for this mortar composition, employing the 70° pre-set angular shear test mold, are illustrated in Figure 9a. This Figure illustrates the mean shear and normal stress values at the fracture surface of the specimen, which are τ = 5.08 MPa and σ = 1.85 MPa, respectively. In Figure 9b, a single set of MC parameters is given for the combined material phases of mortar specimens using the MC constituent model. In other words, in Figure 9b,c, the values of c = 4.1 MPa and ϕ = 14.88° are given for the mortar containing coated-EVA with a coating thickness of 1 mm with a 66% replacement ratio. The material properties employed in the FE analysis are enumerated in Table 3.

**Figure 12:**
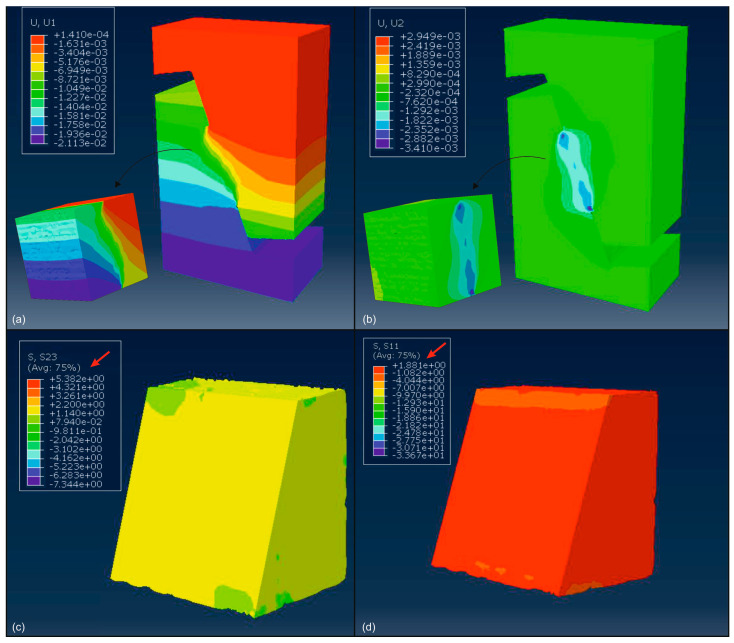
Some results from the FE analysis: (**a**) vertical displacement, (**b**) Out-of-plane displacement, (**c**) shear stress, (**d**) direct stress.

As illustrated in Figure 12, the FE analysis yielded several notable outcomes. As can be seen from Figure 12c,d, as a result of the FE numerical simulation, the maximum shear and normal stress values at the 70° diagonal deformation surface of the specimen were obtained as τ = 5.382 MPa and σ = 1.881 MPa, respectively. The τ and σ results obtained with the same c and ϕ values defined for the aggregate and mortar phases with the MC constitutive model parameters defined in Figure 9b,c (*R_R_* = 66%, *C_t_* = 1 mm) and given in Table 3 are very close to the experimental results of the pre-set angular shear test. Consequently, the computer simulations of the mechanical behavior of mortar containing coated-EVA (*R_R_* = 66%, *C_t_* = 1 mm) demonstrate that the mortar material can be defined as an integrated composite material depending on the coated-EVA content and can be simulated with the MC constitutive model parameters given in Figure 9b,c.

The splitting tensile strength test (ASTM C496 [64]) was performed on three mortar specimens (50 × 100 mm) prepared with the coated-EVA aggregate (*R_R_* = 66%, *C_t_* = 1 mm). The obtained strength values ($\sigma_{t}=4.12\pm 0.2$ MPa) were reduced by applying a factor of 0.8 in order to approximate the uniaxial tensile strength in a practical sense (e.g., [61]), and this adjusted value was adopted as the tension cutoff parameter (Table 3). Furthermore, nine cubic specimens (50 mm) taken from the same mixture were subjected to shear testing in accordance with ASTM D5607 [65], yielding average apparent values of *c*, ϕ, and ψ as 4.32 MPa, 15.47°, and 7.62°, respectively. The dilation angle value was incorporated as an MCP constitutive model parameter in the FE simulation of the specimen (*R_R_* = 66%, *C_t_* = 1 mm) (Table 3). The close agreement between the shear test results obtained using ASTM D5607 and those employed in this study validates the adopted testing approach. In addition, using the material parameters presented in Table 3, the mechanical response of the specimen (*R_R_* = 66%, *C_t_* = 1 mm) was simulated through FE analysis, and the stress results obtained were compared with the experimental results, thereby providing numerical validation of the test outcomes.

## 6. Statistical Evaluation

Regression analyses are carried out for each response to ascertain the effects of the factors on the parameters, using the test results obtained from the compressive strength, oven dry density, thermal conductivity, flexural strength, water absorption, porosity, sorptivity, flow table, and pre-set angular shear tests. The regression models obtained are necessary for the RSM (response surface method) optimization analyses, and they also demonstrate the combined effects of parameters and the contribution of factors to the responses. In this analysis, the dependent variables were the measured properties, such as the compressive strength (σ), flexural strength (f), thermal conductivity (κ), oven dry density ($\delta_{od}$), flow diameter ($D_{f}$), water absorption (WA), porosity (n), initial sorptivity ($S_{I}$), secondary sorptivity ($S_{S}$), cohesion (c), angle of friction (ϕ), and maximum shear stress (τmax). The independent variables were the coated-EVA replacement ratio ($R_{R}$) and the average coating thickness ($C_{t}$). A GLM-ANOVA analysis was conducted at a 95% confidence interval to ascertain the statistical significance of the factors ($R_{R}$ and $C_{t}$) on the responses. Table 4 shows the results of this analysis. As demonstrated in Table 4, the influence of the factors on the responses of σ, f, κ, $\delta_{od}$, $D_{f}$, n, $S_{I}$, $S_{S}$, c, ϕ, and τmax (with the exception of the impact on WA) is statistically significant at the 95% confidence interval, as evidenced by *p*-values less than 0.05. In consequence, the regression models established the correlations between the independent parameters and the responses. In contrast, the combined effect of independent parameters on the response of WA was found to be significant, as evidenced by the regression models. Power transformations were employed to enhance the models of the responses. Firstly, the cubic model was incorporated into the regression study for each response. Subsequently, a stepwise-backward selection algorithm was employed at a 0.05 confidence level with the objective of reducing the complexity of the model. Consequently, terms for the dependent variables that were not statistically significant were removed from the regression models using ANOVA and t-statistics for the null hypothesis for each regression model. Subsequently, regression equations were constructed utilizing the significant terms. Furthermore, Table 4 illustrates the proportion of the independent variables that contribute to the responses, thereby demonstrating the efficacy of the factors on the obtained responses.

In calculating the percentage contributions in Table 4, the Eta-Square Technique (see, for example, Refs. [66,67]) was employed to estimate the effectiveness of factors on the dependent variables. Thus, the efficacy of each factor is specified. Table 4 illustrates the cumulative percentage contribution of the factors of $R_{R}$ and *C_t_*, with the error term representing 100%. The results of the experiments and data analysis, as presented in Table 4, indicate that the independent variable of *C_t_* has the most significant overall impact on the responses, with the exception of the parameters of f, κ, and $\delta_{od},$ where the effectiveness of *R_R_* is more pronounced.

In order to ensure statistical reliability and to ensure that the scope of this study is consistent with the principles of the Design of Experiments (DOE), a full factorial experimental design was applied within the specified parameter ranges. The outputs of the optimization process, obtained from statistical modeling, were verified by conducting additional experimental tests under conditions that were predicted to be optimal. In the comparison made, a deviation of up to 5% was observed between the statistically predicted values and the experimental results. The low deviation rate serves to confirm the robustness and predictive power of the statistical model that has been utilized, thereby demonstrating its validity within the relevant design range.

## 7. Pareto-Efficient Boundaries

In this study, multi-objective optimization is employed to specify the optimal solution sets, with the factors of *R_R_* and *C_t_* and the dependent parameters σ, f, κ, $\delta_{od}$, $D_{f}$, WA, n, $S_{I}$, $S_{S}$, c, ϕ, and τ_max_ taken into account. The optimization procedures were conducted separately for each parameter, with the results presented in Table 5. As there are multiple objective functions given in Table 5, the optimization using RSM, as described by Whitcomb (2004) [68], was employed using multivariate models (e.g., [69]). RSM is a robust optimization technique that is employed globally in engineering applications.

To verify the robustness and predictive validity of the regression models used in the RSM optimization, several statistical diagnostics were performed. Each of the nine response variables was modeled independently using multiple regression with two primary factors. For all models, the correlation coefficients (R) exceeded 0.9, and the difference between adjusted R^2^ and R^2^ remained below 0.2, indicating model consistency. To verify the reliability of the fitted models, variance inflation factor (VIF) values were examined for all factors included in Equations (1)–(9), and all results were found to be within acceptable limits, confirming that multicollinearity did not affect the statistical validity. Adequate precision values were greater than four, confirming sufficient signal-to-noise ratios. Residual analysis demonstrated that model errors were predominantly normally distributed, as confirmed by normal probability plots. Predicted-versus-actual plots showed strong linear correlations, supporting model validity. Power transformation techniques were applied, and optimal λ values were determined using Box–Cox plots to ensure homoscedasticity and normality of variance. Further diagnostic checks, including Cook’s distance, leverage values, and DFFITS statistics, confirmed that no data points exerted undue influence on model outcomes. These results collectively confirm the statistical soundness of the models, justifying their use in response surface optimization.

The following regression equations were obtained from the regression analysis for the responses of σ, f, κ, $\delta_{od}$, $D_{f}$, WA, n, $S_{I}$, and $S_{S}$:

(1) $$\sigma^{1.09}=59.0597-2.1545\;R_{R}+27.1904\;C_{t}+0.0328\;R_{R}^{2}-5.4465\;C_{t}^{2}-0.0002\;R_{R}^{3}+0.3207\;C_{t}^{3}$$

(2) $$\begin{array}{l} f^{0.62}=4.2431-0.0413\;R_{R}+0.9708\;C_{t}+0.0080\;R_{R}\;C_{t}+0.0002\;R_{R}^{2}-0.3486\;C_{t}^{2}-0.00002\;R_{R}^{2}\;C_{t}\\ \;\;\;\;\;\;\;\;\;\;\;\;-0.00065\;R_{R}\;C_{t}^{2}+0.0294\;C_{t}^{3} \end{array}$$

(3) $$\kappa^{0.73}=1.0753-0.0090\;R_{R}+0.0368\;C_{t}+0.0007\;R_{R}\;C_{t}+0.00002\;R_{R}^{2}-0.0041\;C_{t}^{2}-0.00007\;R_{R}\;C_{t}^{2}$$

(4) $$\delta_{od}^{1.45}=2.8020-0.0210\;R_{R}+0.0315\;C_{t}+0.0044\;R_{R}\;C_{t}-0.0057\;C_{t}^{2}-0.0004\;R_{R}\;C_{t}^{2}$$

(5) $$D_{f}^{3}=1801.0046-55.6267R_{R}+1850.2191C_{t}+2.3119\;R_{R}^{2}-645.1862\;C_{t}^{2}-0.0173\;R_{R}^{3}+52.2009\;C_{t}^{3}$$

(6) $$WA^{-0.4}=0.3863-0.0020\;R_{R}-0.0007\;C_{t}+0.0004\;R_{R}\;C_{t}-0.0003\;C_{t}^{2}-0.00004\;R_{R}\;C_{t}^{2}$$

(7) $$\begin{array}{l} n^{1.19}=39.7553+0.5707\;R_{R}+4.1982\;C_{t}+0.0567\;R_{R}\;C_{t}-0.0040\;R_{R}^{2}-4.1916\;C_{t}^{2}-0.0035\;R_{R}^{2}\;C_{t}\\ \;\;\;\;\;\;\;\;\;\;\;+0.0523\;R_{R}\;C_{t}^{2}+0.00005\;R_{R}^{3}+0.4923\;C_{t}^{3}+0.0004\;R_{R}^{2}\;C_{t}^{2}-0.0077\;R_{R}\;C_{t}^{3} \end{array}$$

(8) $$S_{I}^{1.01}=0.0127+0.0013\;R_{R}-0.0139\;C_{t}+0.0001\;R_{R}\;C_{t}-0.00002\;R_{R}^{2}+0.0023\;C_{t}^{2}-0.0002\;C_{t}^{3}$$

(9) $$S_{S}^{1.55}=0.0001-0.000004\;R_{R}+0.000002\;C_{t}-0.000001\;R_{R}\;C_{t}+0.00002\;C_{t}^{2}$$

where *R_R_* is the strengthened-EVA replacement rate (%), *C_t_* is the average coating-thickness of strengthened-EVA (mm), σ is the compressive strength (MPa), f is the flexural strength (MPa), κ is the thermal conductivity (W/mK), $\delta_{od}$ is the oven dry density (gr/cm^3^), $D_{f}$ is the flow diameter (cm), WA is the water absorption (%), n is the porosity (%), $S_{I}$ is the initial sorptivity (mm$/\sqrt{sec}$), and $S_{S}$ is the secondary sorptivity (mm/$\sqrt{sec}$) values.

**Figure 13:**
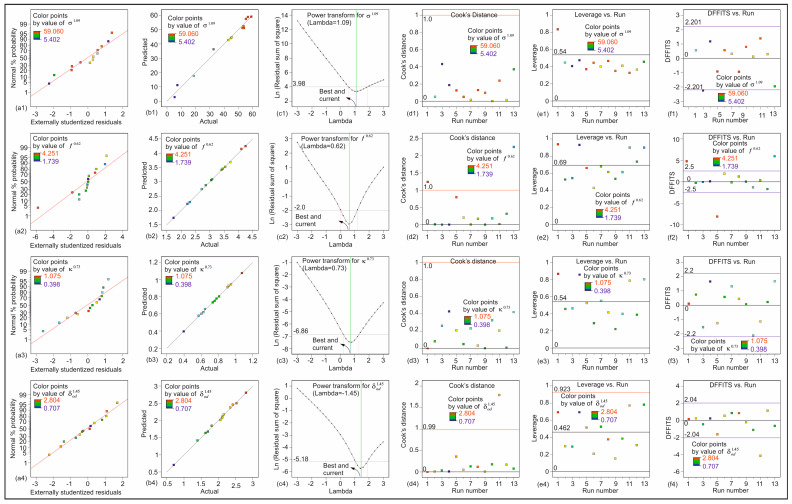
Statistical analysis of the models in Equations (1)–(4): (**a1**–**a4**) normal probability plots of residuals for the models, (**b1**–**b4**) predicted and actual values, (**c1**–**c4**) transformation analysis of Box–Cox, (**d1**–**d4**) Cook distance demonstration, (**e1**–**e4**) leverage variation in the point of design, and (**f1**–**f4**) the values of DFFITS.

Figure 13 presents a statistical analysis of the models in Equations (1)–(4), which is given as a representative example. Figure 13(a1–a4) illustrate the normal probability graph of the residuals for the models in Equations (1)–(4). Figure 13(a1–a4) demonstrate that the errors are normally distributed, as evidenced by the predominantly linear distribution of the residuals. Figure 13(b1–b4) illustrate a linear relationship between the predicted and actual values. Figure 13(c1–c4) present the transformation analysis of Box–Cox for the responses in Equations (1–4), respectively. Figure 13(d1–d4) illustrate the impact of the data on the regression models in Equations (1)–(4) through the Cook’s distance demonstration. This metric demonstrates the extent of variation in the predicted responses if experiments are not incorporated into the regression model construction. The Cook distances indicate that the results are not significantly divergent from the remaining data points, suggesting that the modeling is accurate and that there are no registration errors. Figure 13(e1–e4) illustrate the variation in leverage at the design points. It can be observed that there are no design points in close proximity to a value of one, with the majority situated between twice the average leverage and below this threshold. This suggests that the outcomes under consideration exert a considerable influence on the regression models. The discrepancy in fits (DFFITS values) is illustrated in Figure 13(f1–f4), which demonstrate the disparity in predicted outcomes for each design point in the absence of regression model construction. As illustrated in Figure 13(f1–f4), the points of design fall into the reasonable range.

In the process of RSM optimization, the objective functions using the objectives given in Table 5 are optimized while varying the factors of $R_{R}$ and *C_t_* using the regression models. The desirability functions ($d_{i}$) given below are characterized for the values in Table 5 and were used to simultaneously optimize the responses using the previously underlined process (e.g., [66,67]). In consideration of the aforementioned functions of desirability within the specified range of ${0\leq d}_{i}\leq 1$, a singular response (D) is derived, which amalgamates the functions of individual desirability. Subsequently, the composite response is maximized in accordance with the objectives delineated in Table 5. A composite response of zero indicates that the outcome falls outside the desired range. A composite response of one indicates that the objectives have been fully achieved. All dependent parameters with defined targets, as outlined in Table 5, are incorporated into a singular response in accordance with the methodology proposed by Myers et al. (2009) [70], wherein an equal importance weight is assigned to each target in Table 5. In the desirability-based multi-objective optimization process conducted in this study, all response variables were assigned a uniform importance level (weight = 3) to maintain a neutral baseline for comparative analysis. This approach was adopted due to the absence of a priori information regarding the relative criticality of each parameter within the target application. It is acknowledged that, in practice, certain parameters (e.g., compressive strength, water absorption, or sorptivity) may be deemed more critical than others. However, the proposed optimization process should be reproduced by designers and readily adjusted to satisfy the desirability weights and importance levels of individual responses based on their specific design criteria or performance expectations. Consequently, the presented framework functions as a generalizable and adaptable decision-making tool for material design optimization.

In order to obtain the optimal bounds, the parameters of σ, f, κ, $\delta_{od}$, $D_{f}$, WA, n, $S_{I}$, $S_{S}$ c, ϕ, and τmax are utilized as outputs, while the factors of $R_{R}$ and $C_{t}$ are considered as inputs. It should be noted that the solution reached may not necessarily be the globally optimized and most efficient solution. In order to obtain the most efficient solution, it is necessary to refer to the solution at the relevant boundary when proposing a combination. In this regard, the pursuit of an optimal efficient frontier in a multi-objective optimization problem is of paramount importance, as postulated by Hou et al. (2009) [71]. Although the combinations of input–output variation represent a feasible type of solution, as previously stated, the solution set must be on the line of the frontier, which is known as an efficient solution set. Consequently, 20 sets of optimization analyses were performed based on RSM, using the definition of parameters outlined in Table 5, with the aim of obtaining the efficient design of materials using the global frontier.

The outcomes of multi-objective optimization employing RSM are attained as the optimal result of a data set. A series of optimization analyses was conducted in accordance with the objectives of the responses specified in Table 5. Within the solution set curves, there is a curve where the Di values are maximized, which is defined as the boundary curve. In this study, in addition to the parametric optimization objectives given in Table 5, two different boundaries were obtained for the maximization and minimization of oven dry density. The boundaries containing the solution sets obtained as a result of maximizing the Di values within the parametric optimization objectives are specified. Therefore, any point on these two curves is defined as the effective solution. The region between these two boundaries is defined as the feasible region, as it represents all other possible optimal solution sets of oven dry density. The highest and lowest values above the maximized and minimized optimal limit functions are referred to as global optima. These global optima are defined as the most optimal outcomes possible. Accordingly, the solution that yields the maximum D_i_ value among the suite of solutions derived from a series of optimization analyses, wherein the oven dry density is maximized and the objectives of other objectives are altered, can be designated as the global optimum. As the D_i_ values within the feasible region are minimal, the region between the boundaries is designated as the feasible optimum region, given its absence of a high-level optimization solution, in comparison to the solution sets located on the boundary curves. Consequently, the decision maker is empowered to select from the outcomes presented by a design block, thereby facilitating the resolution of a multi-objective optimization problem. The decision maker can ascertain the replacement ratio and coating thickness of coated-EVA to be utilized in mortar. This is contingent on the target compressive strength, flexural strength, and thermal conductivity or oven dry density values.

**Figure 14:**
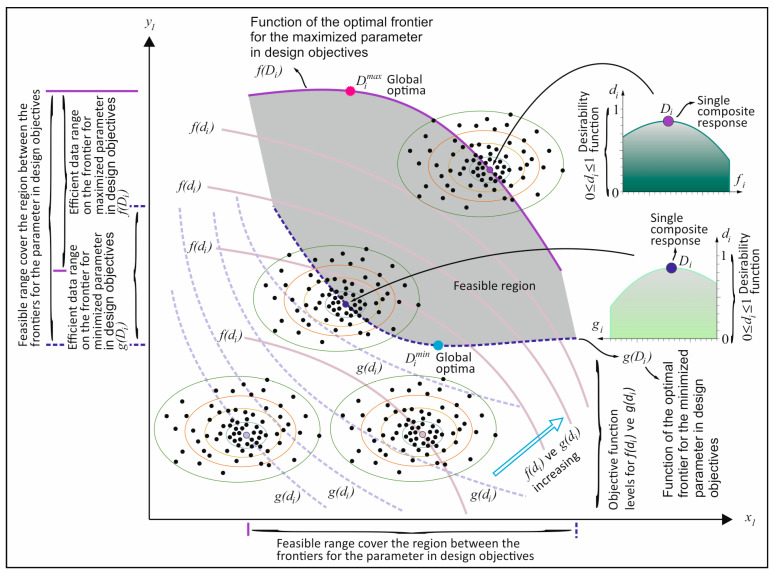
Schematic illustration of optimum frontier curves developed in this paper.

A variety of methodologies are employed in the resolution of multi-objective optimization problems; one such methodology is the Pareto-optimal method [72]. The rationale behind employing Pareto-optimization in multi-objective optimization problems is that these problems do not possess a single global optimum, and the decision maker has multiple alternatives. As demonstrated in Figure 14, the outcomes of multi-objective optimization employing RSM are attained as the optimal result of a data set. A series of optimization analyses was conducted in accordance with the objectives of the responses specified in Table 5. Within the solution set curves, there is a curve where the Di values are maximized. This curve is defined as the boundary curve and is shown in Figure 14. In this study, in addition to the parametric optimization objectives given in Table 5, two different boundaries were obtained for the maximization and minimization of oven dry density. A schematic representation of the methodology employed to derive these boundaries is provided in Figure 14. The boundaries containing the solution sets obtained as a result of maximizing the Di values within the parametric optimization objectives are shown schematically in Figure 14, and any point on these two curves is defined as the effective solution. The purple boundary delineates the solution set where oven dry density is maximized, while the blue boundary delineates the solution set where oven dry density is minimized. The region between these two boundaries is defined as the feasible region, as it represents all other possible optimal solution sets of oven dry density. The highest and lowest values above the maximized and minimized optimal limit functions are referred to as global optima. These global optima are defined as the most optimal outcomes possible. In summary, the solution that yields the maximum Di value among the suite of solutions derived from a series of optimization analyses, wherein the oven dry density is maximized and the objectives of other objectives are altered, can be designated as the global optimum. As the Di values within the feasible region are minimal, the region between the boundaries is designated as the feasible optimum region, given its absence of a high-level optimization solution, in comparison to the solution sets located on the boundary curves. Consequently, the decision maker is empowered to select from the outcomes presented by a design block, thereby facilitating the resolution of a multi-objective optimization problem. The decision maker can ascertain the replacement ratio and coating thickness of the coated-EVA aggregate to be utilized in mortar. This is contingent on the target compressive strength, flexural strength, thermal conductivity, or oven dry density values.

**Figure 15:**
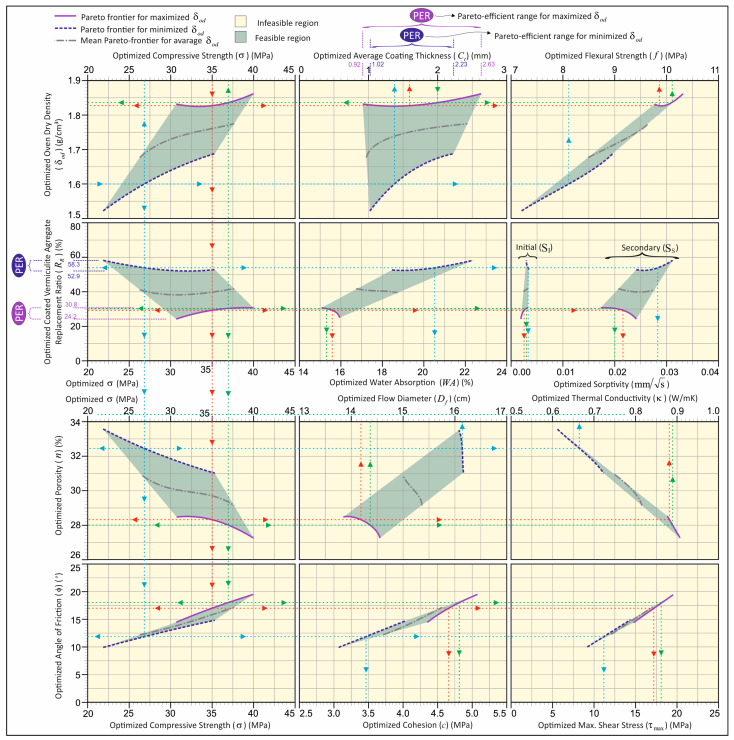
Optimal efficiency analysis based on the dual optimization of $\delta_{od}$.

Figure 15 demonstrates that Pareto-efficiency analysis can be conducted with optimal interaction between parameters and that multi-objective efficiency analysis effectively evaluates with dual optimization. Accordingly, for example, when searching for the most efficient values of the optimum vermiculite aggregate replacement (*R_R_*) and the optimum average coating thickness (*C_t_*), efficiency analysis can be performed in material design by graphically obtaining the solution that corresponds to the desired response value on the Pareto-frontiers.

In accordance with the design chart illustrated in Figure 15, the optimal solution sets, contingent on the specified optimization objectives, can be derived. The efficient frontiers for the maximized and minimized variations are illustrated in Figure 15, delineating the upper and lower limitations of the feasible region, respectively, as indicated by solid and dashed lines. It is only possible to obtain efficient solutions if they lie on the lines of frontiers. These limitation lines are employed to specify the solutions of *R_R_* and *C_t_* in the design of lightweight mortar produced with the efficient coated-EVA using the minimum and maximum utilizations of *R_R_* and *C_t_* to achieve an efficient design.

The projection of the feasible solution domain on the axis of the graph in Figure 15 identifies the boundary of the feasible region in that axis. The feasibility intervals indicate the existence of an optimal solution within the specified range. In the event that the optimal solution is located on the frontier, this suggests that an efficient solution is also implied. As illustrated in Figure 1, the analysis of efficiency within the efficient frontier can be facilitated through the interaction between parameters (see Examples 1, 2, and 3). Moreover, these illustrations underscore the pivotal role of frontiers in attaining an efficient solution set. The projection distance PER (Pareto-efficient range) on the graph axis is defined as the efficient interval.

The results of the efficiency analysis presented in Figure 15 indicate that, in the context of the maximum oven dry density criterion for lightweight mortars, the optimal usage ratio of coated-EVA ranges from 24.2% to 30.8%, while the coating thickness varies between 0.92 and 2.63 mm. Utilizing the minimum oven dry density criterion, the optimal usage ratio was determined to range from 52.9% to 58.3%, with a coating thickness ranging from 1.02 to 2.23 mm. The purple solid boundary line shown in Figure 14 indicates the solution set with the highest oven dry density, while the blue dashed boundary line indicates the solution set with the lowest oven dry density. The region delineated by these boundaries is designated as the feasible region, as it encompasses all potential optimal solution sets for oven dry density. When targeting maximum oven dry density, the purple solid line in the relevant graphs will be used as a reference. Conversely, when targeting minimum oven dry density, the blue dashed boundary line will be used as a reference. For instance, if a lightweight mortar design with minimum density is desired, the blue dashed line should be used as the reference in all graphs presented in Figure 15. Subsequent to determining the target value along the axis of the graph, all pertinent design parameters can be obtained by the intersection with this line.

As demonstrated in Figure 15, there are three distinct application examples. In the initial example (illustrated by the red line), the optimal replacement ratio of the coated-EVA for the design of a lightweight mortar with a targeted maximum dry density and 35 MPa compressive strength is determined to be 29.5%, and the optimal coating thickness is determined to be 1.6 mm. Additionally, by using the Pareto-frontier curves, the parameters of $\delta_{od}$, f, κ, $D_{f}$, WA, n, $S_{I}$, $S_{S}$ c, ϕ, and τmax are determined to be approximately 1.83 gr/cm^3^, 9.85 MPa, 0.88 W/mK, 14.2 cm, 15.5%, 28.3%, 0.0025 $\mathrm{mm}/\sqrt{s}$, 0.0215 $\mathrm{mm}/\sqrt{s}$,4.65, 19°, and 15.4 MPa, respectively. In the second example (illustrated with the blue line), the optimal replacement ratio for coated-EVA for a lightweight mortar design with a density value of 1.6 g/cm^3^ was determined to be 54%, and the optimal coating thickness was determined to be 1.2 mm. It is imperative to acknowledge that the density value of 1.6 g/cm^3^ falls within the optimization solution sets where density is minimized.

Consequently, the blue dashed line, representing the Pareto-frontier curves where density is minimized, has been employed in all graphs. Furthermore, utilizing the Pareto-frontier curves σ, f, κ, $D_{f}$, WA, n, $S_{I}$, $S_{S}$ c, ϕ, and τ_max_ were determined to be approximately 26.75 MPa, 8.1 MPa, 0.665 W/mK, 16.1 cm, 20.5%, 32.4%, 0.0035 $\mathrm{mm}/\sqrt{s}$, 0.0282 $\mathrm{mm}/\sqrt{s}$, 3.48, 12°, and 11.25 MPa, respectively. In the third example (illustrated with a green line), it was determined that the optimum replacement ratio of the aggregate should be approximately 30% for a lightweight mortar mixture designed with a maximum density target and using coated vermiculite aggregate with a coating thickness of 2 mm. In addition, by using the Pareto-frontier curves, the parameters of $\delta_{od}$, σ, f, κ, $D_{f}$, WA, n, $S_{I}$, $S_{S}$ c, ϕ, and τmax are determined to be approximately 1.84 gr/cm^3^, 37 MPa, 10.1 MPa, 0.89 W/mK, 14.4 cm, 15.3%, 28%, 0.003 $\mathrm{mm}/\sqrt{s}$, 0.02 $\mathrm{mm}/\sqrt{s}$, 4.8, 18°, and 18.1 MPa, respectively. As demonstrated by the provided examples, the achievement of the targeted Pareto-efficient solution parameter is attainable in the solution steps by commencing the process from the desired/known parameter. In this context, it is possible to modify the solution directions in accordance with the requirements of the user.

**Figure 16:**
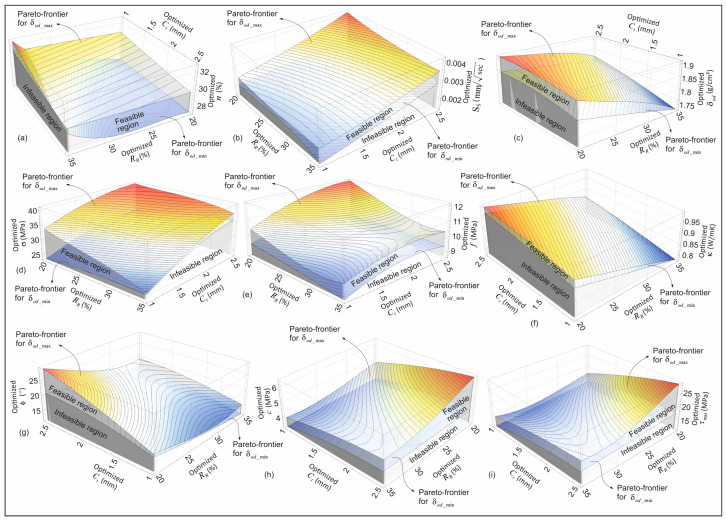
The 3D variation in the Pareto-efficient boundary curves: (**a**–**c**) 3D Pareto-frontier variations in terms of the optimized parameters of *C_t_*, *R_R_*, *n*, *S_S_* and $\delta_{od}$, (**d**–**f**) 3D Pareto-frontier variations in terms of the optimized parameters of σ, *C_t_*, *R_R_* , *n*, f and κ, (**g**–**i**) 3D Pareto-frontier variations in terms of the optimized parameters of ϕ, *C_t_*, *R_R_*, c, τ_max_.

The optimum boundary curves obtained from the Pareto-efficiency analysis (see Figure 15) (the optimum boundary curves obtained as a result of maximizing and minimizing the oven dry density in addition to the optimization objectives given in Table 5) can be explained by three-dimensional (3D) optimum boundary surfaces based on all dependent and independent variables, as shown in Figure 16. In order to provide a clearer and more comparative representation of these 3D optimal boundary surface variations (illustrated as 3D visualizations of the 2D curves given in Figure 15), the same parametric ranges are employed. The region between the 3D optimum boundary surface variations delineated in Figure 16 is designated as the feasible region. As is the case for the 2D optimum boundary curves in Figure 15, in addition to the optimization objectives given in Table 5, the optimum solutions for the cases of maximizing and minimizing oven dry density are also valid for the 3D optimum boundary surface variations shown in Figure 16. This visualization technique provides a clear representation of the 3D variation in the Pareto-efficient frontiers, thereby facilitating a more discernible understanding of how the feasible region is influenced by the alteration of parameters.

## 8. Limitations and Future Work

The present study has examined some properties of different coating thicknesses. However, a comprehensive investigation of the microstructural changes (e.g., porosity gradient and quantification of interfacial transition zone (ITZ) phases) associated with coating thicknesses has been limited. It is imperative to recognize the significance of microstructural characterizations in facilitating a comprehensive understanding of the fundamental mechanisms that influence material performance. These characterizations are instrumental in establishing the current findings on a more robust foundation, thereby ensuring the reliability and validity of the research outcomes. Consequently, subsequent studies will utilize sophisticated microscopic methodologies to undertake a comprehensive examination of the impact of coating thicknesses on microstructural characteristics. This will facilitate a more in-depth and mechanistic elucidation of the effects of the coating process on mechanical performance and durability.

In this study, SEM analyses were limited to qualitative microstructural observations and did not include EDS elemental mapping or quantitative image analysis of features such as ITZ thickness or porosity fraction. While the SEM micrographs provided valuable visual context supporting macro-scale performance results, a comprehensive microstructural characterization was not within the scope of the current work. In future studies, advanced techniques, such as EDS mapping and quantitative SEM image analysis, will be utilized to establish stronger correlations between microstructural parameters and the mechanical behavior of the composite mortar system. In this study, the chemical interactions between the coated aggregates and the cement paste were not investigated using advanced material characterization methods, such as FTIR, XRD, or TGA. While the primary focus was on the mechanical behavior of the mortar systems, we acknowledge that understanding the interfacial chemistry is crucial for a comprehensive evaluation of the composite performance. Future research will incorporate these techniques to explore the possible chemical bonding mechanisms and phase evolutions occurring at the aggregate–matrix interface.

This study did not include rheological measurements, such as yield stress and viscosity, which are important for a deeper understanding of workability and its relation to flow behavior. For this reason, the discussion of fresh-state properties in this manuscript was limited, and the main emphasis was placed on the mechanical performance of mortars and the derivation of constitutive model parameters. Future research should incorporate detailed rheological analyses to establish stronger correlations between particle morphology, surface characteristics, workability, and mechanical behavior.

Durability tests in this study were conducted under limited and controlled laboratory conditions. Therefore, critical environmental effects, such as freeze–thaw cycles, thermal variations, and chloride exposure, which are commonly encountered in real-world applications, were not considered. Recognizing the importance of these factors in long-term performance, future work will include comprehensive durability evaluations to investigate how such environmental stressors influence the mechanical integrity and chemical stability of the coated aggregate system. In this study, mechanical and physical tests were performed only after a 28-day curing period. While the 28-day age serves as a standard reference point for evaluating the performance of cementitious composites, time-dependent behaviors, such as early-age strength development and long-term durability characteristics, were not addressed. Future work will focus on the detailed investigation of early-age mechanical properties and the evolution of long-term durability parameters (e.g., freeze and thaw resistance, shrinkage, sulfate resistance) of mortars incorporating coated aggregates under varied environmental exposures. Although this study highlights the potential eco-efficiency of the multilayer aggregate coating technique, it does not include a quantitative environmental or sustainability analysis. Metrics such as embodied energy, carbon footprint, and other life cycle indicators were not evaluated. The current research focused primarily on laboratory-scale characterization of mechanical and microstructural behavior. Future studies will incorporate comprehensive environmental assessments based on life cycle analysis (LCA) to better quantify the sustainability implications of the proposed coating process in relation to its mechanical performance benefits.

In the presented 3D FE simulation, the dilation angle ψ in the Mohr–Coulomb constitutive model was set to zero. This assumption is supported by the relatively low internal friction angle (φ = 14.88°) obtained for the coated-EVA mortar (Table 3) and aligns with conventional implementations whereby low-friction, moderate-cohesion materials exhibit minimal dilation. No significant volumetric deformation was observed in the experimental shear tests, and the simulation results closely matched the stress responses without invoking dilation. While dilation can be important in granular composites with higher friction or densification, its neglect here is technically justified. Future studies involving higher φ values or more densely packed materials will consider calibrating ψ based on direct measurements from triaxial or drained shear tests.

## 9. Conclusions

The following conclusions can be drawn from the results of this study:

- A multilayer coating strategy applied to EVA aggregates has demonstrated notable improvements in both mechanical and durability properties of lightweight mortar, validating the effectiveness of the proposed methodology.
- A Pareto-efficiency-based multi-objective optimization approach enabled the identification of optimal coating thickness and replacement ratio ranges, offering a practical tool for performance-based aggregate design.
- The modified aggregates enhanced compressive strength and shear resistance significantly, complying with lightweight aggregate specifications, leading to improved strength-to-weight efficiency.
- The coated-EVA aggregates contributed to reduced apparent porosity and water absorption, indicating enhanced microstructural integrity and the durability performance of the mortar.
- The mechanical response of the mortars was consistent with composite behavior, and the results confirmed that the material could be effectively modeled using the Mohr–Coulomb constitutive framework.
- Compared to conventional uncoated-EVA usage, the proposed coated-EVA process enables the development of lightweight structural mortars that meet higher strength and thermal insulation requirements simultaneously.

## Figures and Tables

**Table 1:** Physical properties of coated-EVA.

	Average Coating Thickness (mm)		
	1	2	3
Loose bulk density (kg/m^3^)	379	373	360
Specific gravity	1.75	1.78	1.81
Water absorption (%)	24	21	18

**Table 2:** Material quantities used for lightweight mortar (kg/m^3^).

Mixture ID	Replacement Ratio (*R_R_*)	Coating Thickness (*C_t_*)	w/c	Cement	Water	River Sand	*C_t_* = 0	*C_t_* = 1	*C_t_* = 2	*C_t_* = 3
33%EV-0	33	0	0.5	550	275	967	40			
66%EV-0	66	0	0.5	550	275	491	79			
100%EV-0	100	0	0.5	550	275	0	120			
33%EV-1	33	1	0.5	550	275	967		315		
66%EV-1	66	1	0.5	550	275	491		630		
100%EV-1	100	1	0.5	550	275	0		954		
33%EV-2	33	2	0.5	550	275	967			320	
66%EV-2	66	2	0.5	550	275	491			641	
100%EV-2	100	2	0.5	550	275	0.00			971	
33%EV-3	33	3	0.5	550	275	967				326
66%EV-3	66	3	0.5	550	275	491				651
100%EV-3	100	3	0.5	550	275	0				987

**Table 3:** Material properties used in FE analysis.

Materials	Constitutive Models	*ρ*(*t*/*mm*^3^) × (10^−6^)	*E* (GPA)	ν	*ϕ* (°)	*ψ* (°)	*c* (MPA)	σ_*t*−*cut*_ (MPA)
Upper and lower molds	LE	7.85	210	0.3	-	-	-	-
Cement–silica fume liner	MCP	2.0	20	0.22	14.88	7.62	4.1	3.3
Raw vermiculite (EVA)	MCP	0.00016						
Cement paste	MCP	2.1						

Note 1: LE: linear elastic model, MCP: Mohr–Coulomb plasticity model, E: elastic modulus, ν: Poisson’s ratio, ρ: density, ϕ: friction angle, ψ: dilation angle, c: cohesion, and $\sigma_{t-cut}:$ tension cutoff stress. Note 2: The FE simulation of the specimen (*R_R_* = 66%, *Ct* = 1 mm) was conducted in a manner that reflects the integrated behavior of the MCP constitutive model parameters. Since *c* and ϕ were determined based on the shear test representing the composite behavior, the cement–silica fume liner, raw vermiculite (EVA), and cement paste components were characterized using the equivalent values of E, ν, ϕ, ψ, c, and $\sigma_{t-cut}$. These parameters were defined with equivalent values representing the overall mortar response, thereby enabling the simulation of the composite behavior of the mortar system.

**Table 4:** ANOVA results.

Responses	Variation	Parameters					Significant	PCx (%)	Variables	Source	Statistical Parameters					Significant	PCx (%)
		df	SSx	MSx	F	*p*-value					df	SSx	MSx	F	*p*-value		
σ	$R_{R}$	**3**	**376.06**	**351**	**72.92**	**<0.001**	Yes	18.26	n	$R_{R}$	3	397.97	173.07	12.76	<0.01	Yes	35.29
	$C_{t}$	3	1654.59	551.53	114.58	<0.001	Yes	80.34		$C_{t}$	3	181.16	60.39	4.45	<0.01	Yes	55.88
	Error	6	28.88	4.81				1.40		Error	6	81.37	13.56				8.82
f	$R_{R}$	3	35.719	16.252	166.13	<0.001	Yes	53.69	$S_{I}$	$R_{R}$	3	0.0009	0.0005	291.70	<0.001	Yes	33.21
	$C_{t}$	3	30.218	10.073	102.96	<0.001	Yes	45.42		$C_{t}$	3	0.0018	0.0006	343.22	<0.001	Yes	66.42
	Error	6	0.587	0.10				0.88		Error	6	0.0000	0.0000				0.37
κ	$R_{R}$	3	0.508	0.1983	355.13	0.01	Yes	83.29	$S_{S}$	$R_{R}$	3	0.00001	0.000001	20.20	<0.01	Yes	30.42
	$C_{t}$	3	0.0985	0.03284	58.81	0.05	Yes	16.15		$C_{t}$	3	0.00002	0.00001	103.73	<0.001	Yes	69.53
	Error	6	0.0034	0.00				0.56		Error	6	0.00000	0.00000				0.04
$\delta_{od}$	$R_{R}$	3	0.762	0.33962	21.98	<0.001	Yes	60.93	ϕ	$R_{R}$	3	214.08	116.61	37.99	<0.001	Yes	45.38
	$C_{t}$	3	0.396	0.13192	8.54	0.01	Yes	31.66		$C_{t}$	3	239.21	79.74	25.97	<0.001	Yes	50.71
	Error	6	0.0927	0.01545				7.41		Error	6	18.42	3.07				3.90
$D_{f}$	$R_{R}$	3	16.374	5.461	20.28	<0.002	Yes	41.79	c	$R_{R}$	3	12.69	7.71	86.59	<0.001	Yes	41.70
	$C_{t}$	3	21.196	7.066	26.24	<0.001	Yes	54.09		$C_{t}$	3	17.21	5.74	64.45	<0.001	Yes	56.55
	Error	6	1.615	0.269				4.12		Error	6	0.53	0.09				1.75
WA	$R_{R}$	3	956.10	445.30	4.13	0.06	No	24.71	$\tau_{\max}$	$R_{R}$	3	263.72	142.29	34.59	<0.001	Yes	47.17
	$C_{t}$	3	758.00	252.70	2.34	0.17	No	57.65		$C_{t}$	3	270.71	90.24	21.94	<0.001	Yes	48.42
	Error	6	647.20	107.90				17.65		Error	6	24.68	4.11				4.41

Note: df: degree of freedom, SSx: sum of square, MSx: mean square, PCx: contribution.

**Table 5:** Parameters in multi-objective optimization.

Names		Goal	Lower Limit	Upper Limit	Units
**Factors**	$R_{R}$	is in range	0	100	%
	$C_{t}$	is in range	0	7	
**Responses**	σ	min., max. or targeted	4.7	42.17	MPa
	f	is in range	2.44	10.32	MPa
	κ	minimize	0.283	1.105	W/mK
	$\delta_{od}$	minimize or maximize	0.788	2	g/cm^3^
	$D_{f}$	is in range	10.33	16.17	cm
	WA	is in range	10.84	66.02	%
	n	is in range	22.08	51.99	%
	$S_{I}$	is in range	0.0036	0.058	$\mathrm{mm}/\sqrt{s}$
	$S_{S}$	is in range	0.0019	0.0062	$\mathrm{mm}/\sqrt{s}$
	ϕ	maximize	1.76	21.1	Degree
	c	maximize	0.79	6.01	MPa
	$\tau_{\max}$	maximize	0.93	22.02	MPa

## Data Availability

The original contributions presented in this study are included in this article; further inquiries can be directed to the corresponding author.
